# N-acetylcysteine lacks universal inhibitory activity against influenza A viruses

**DOI:** 10.1186/1477-5751-10-5

**Published:** 2011-05-09

**Authors:** Mutien-Marie O Garigliany, Daniel J Desmecht

**Affiliations:** 1 Department of Pathology, Faculty of Veterinary Medicine, University of Liège, Belgium

## Abstract

N-acetylcysteine (NAC) has been recently proposed as an adjuvant therapeutic drug for influenza pneumonia in humans. This proposal is based on its ability to restrict influenza virus replication *in vitro *and to attenuate the severity of the disease in mouse models. Although available studies were made with different viruses (human and avian), published information related to the anti-influenza spectrum of NAC is scarce. In this study, we show that NAC is unable to alter the course of a fatal influenza pneumonia caused by inoculation of a murinized swine H1N1 influenza virus. NAC was indeed able to inhibit the swine virus *in vitro *but far less than reported for other strains. Therefore, susceptibility of influenza viruses to NAC appears to be strain-dependent, suggesting that it cannot be considered as a universal treatment for influenza pneumonia.

## Introduction

About 10 percent of the human population is affected by influenza annually and several pandemic episodes have occurred throughout recorded history [1]. This context explains why continued efforts are made to identify new therapeutic molecules. Among these, N-acetylcysteine (NAC), which is commonly used for its mucolytic activity in humans, was shown to inhibit influenza virus both in mouse models, alone or in combination, with the A/PR/8 strain [2,3], and *in vitro*, with H5N1 strains [4]. Recently, NAC treatment was reported to reduce symptoms of influenza-like illness in humans [5] and administration of the dose of 100 mg/kg supposedly contributed to the success of the treatment of a patient infected with the 2009 pandemic H1N1 virus [6].

In order to determine whether these successful results can be extrapolated to other viral strains than A/PR/8 and H5N1 strains, the effect of NAC on the clinical course and outcome of experimental influenza was assessed in mice inoculated with a lethal dose of our murinized swine H1N1 influenza strain [7]. In spite of a significant but very partial anti-influenza effect *in vitro*, neither percent survival nor body weight loss were altered by NAC treatment *in vivo*, suggesting that NAC-susceptibility of influenza A viruses is strain-dependent.

## Methods

### *In vivo *study

Two groups of ten 8-wk old female CD-1 mice were intranasally inoculated with 10 MLD50 of murinized A/swine/Iowa/4/1976 (H1N1) virus [7]. The first group received 100 mg/kg NAC (Sigma) daily by gavage, from day 1 to day 7 post-infection (pi), while the second received the vehicle only. Clinical status, body weight (BW) and mortality were recorded daily up to day 14 pi. Challenge studies were approved by the Belgian Council for Laboratory Animal Science, under the guidance of the Institutional Animal Care and Use Committees of the University of Liège.

### *In vitro *study

Near confluent Vero cells (ATCC CCL-81) were infected at a multiplicity of infection of 0.01 with a Vero cell-adapted variant of the A/swine/Iowa/4/1976 (H1N1) virus in 6-well plates. One hour after infection, fresh DMEM supplemented with 0.2% bovine serum albumin (Invitrogen), 2 μg/ml TPCK-treated trypsin (Sigma), and either 0, 0.5, 1.5 or 2.5 mg/ml of NAC (Sigma) was added onto the cells. Culture supernatants were harvested 48 h after infection and titrated by standard plaque assays.

## Results and discussion

**Figure 1:**
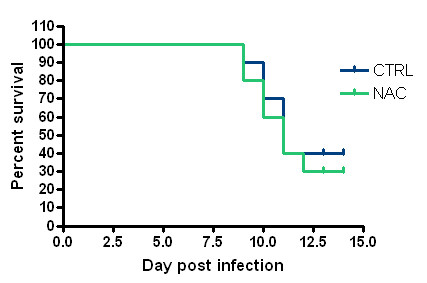
**Kaplan-Meier survival analysis after H1N1 virus inoculation in mock- and N-acetylcysteine-treated mice (n = 10)**.

**Figure 2:**
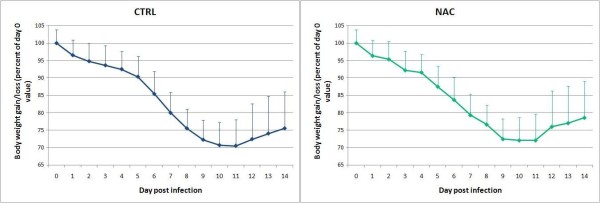
**Effect of mock and N-acetylcysteine oral treatment on body weight course after inoculation of 10 DL50 of H1N1 virus in mice**. Means ± SD (n = 10).

All mice became obviously sick from day 5 pi onward, with lethargy, ruffled coat and respiratory distress. Percent survival and mean survival time (Figure 1) were not different between NAC and control groups (p > 0.05, Kaplan-Meier analysis). Moreover, course and amplitude of BW loss (Figure 2) were similar (p > 0.05, ANOVA). Altogether, the results therefore show that a daily dose of 100 mg/kg NAC did not confer protection against influenza disease in our experimental conditions. The dose of NAC given here (100 mg/kg) is typically that used for high dose NAC treatment of human severe influenza pneumonia in recent clinical trials [6]. Some of the former studies that have enlighted the protective efficacy of NAC in mouse models have used an oral dose of 1000 mg/kg, which suggests that the dose given here is not sufficient. However, 100 mg/kg NAC is already considered as a high dose in humans, and 1000 mg/kg is obviously unrealistic for humans because too close to the LD50 [2,3,8,9]. The fact that a dose of NAC close to that used here drastically diminished mouse lung damages in a diesel-enhanced influenza pneumonia [10] whereas it did not work here rather suggests that the use of different virus strains results in different susceptibilities to NAC *in vivo*.

**Figure 3:**
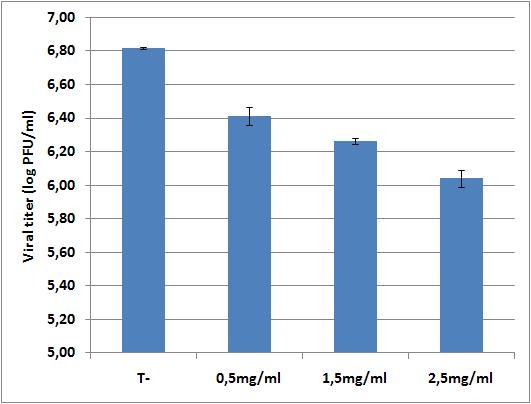
**Effect of N-Acetylcysteine on swine H1N1 virus replication in Vero cells**. Vero cells were infected with A/swine/Iowa/4/1976 (H1N1) at a MOI of 0.01. N-acetylcysteine treatment was started 1 hour post-infection and continued up to 48 hours post-infection. Viral titers were determined 48 hours post-infection. Data represent the mean ± SD of two independent experiments.

To examine this hypothesis further, the susceptibility to NAC of our porcine H1N1 strain was assessed *in vitro *by mimicking Geiler and colleagues methodological approach [4]. We also found a dose-dependent inhibition of influenza virus replication by NAC (Figure 3). However, even a very high dose (2.5 mg/ml, about 15 mM) resulted in a ≈ 6-fold reduction of virus yield, which is far less than that observed with H5N1 [4]. Thus, the anti-influenza activity of NAC appears to be strain-dependent as already supposable from previous studies [4]. The porcine A/swine/Iowa/4/1976 (H1N1) strain used here seems more resistant to NAC than the human strains A/PR/8/1934 (H1N1) and A/Hong Kong/8/1968 (H3N2) used by other authors in mouse models [2,3,8,10]. The lack of protection *in vivo *recorded in our experimental conditions is therefore probably associated to a combination of the NAC-resistance phenotype and to the pathotype [7] of the virus strain used.

Overall, the *in vitro *and *in vivo *results gathered here show that susceptibility of influenza viruses to NAC is clearly strain-dependent, which suggests that NAC cannot be considered to be a universal treatment for influenza pneumonia. A systematic testing of anti-influenza activity of NAC should be implemented whenever a new strain emerges.

## Abbreviations

NAC: N-acetylcysteine; TPCK: L-1-tosylamido-2-phenylethyl chloromethyl ketone.

## Competing interests

The authors declare that they have no competing interests.

## Authors' contributions

MMG designed the study, performed the experiments and helped to draft the manuscript. DD initiated the study, participated in the analysis and interpretation of data, and drafted the manuscript. All authors read and approved the final manuscript.
